# Association between ambient fine particular matter components and subsequent cognitive impairment in community-dwelling older people: a prospective cohort study from eastern China

**DOI:** 10.1007/s40520-024-02793-9

**Published:** 2024-07-26

**Authors:** Tao Zhang, Wenfeng Liu, Tao Yang, Yujia Zhai, Xue Gu, Le Xu, Fudong Li, Mengna Wu, Junfen Lin

**Affiliations:** 1 Department of Public Health Surveillance and Advisory, Zhejiang Provincial Center for Disease Control and Prevention, Hangzhou, Zhejiang China; 2 Office, Changshan Center for Disease Control and Prevention, Quzhou, Zhejiang China; 3 Office, Yuhang Center for Disease Control and Prevention, Hangzhou, Zhejiang China

**Keywords:** Cognitive impairment, Fine particular matter components, Cohort studies, Aged, China

## Abstract

**Background:**

Fine particular matter (PM_2.5_) has been associated with dementia, but limited information is available regarding the association between PM_2.5_ components and dementia.

**Aims:**

We aimed to identify the major components of PM_2.5_ that affect cognitive function to further investigate its mechanism of action, and develop a prevention strategy for dementia.

**Methods:**

In this study, we included 7804 participants aged ≥ 60 years recruited from seven counties in Zhejiang province, eastern China. The participants completed the baseline survey between 2014 and 2015, and were followed up until the end of 2020. We adopted single-component robust Poisson regression models for analyses, and estimated relative risks and 95% confidence intervals describing associations between the chemical constituents of PM_2.5_ exposure and incident cognitive impairment in those who were free from cognitive impairment at baseline.

**Results:**

Significantly positive associations were observed between sulfate, nitrate, ammonium, and organic matter in PM_2.5_ and incident cognitive impairment across different exposure periods; the relative risks of 10-year exposure before enrollment ranged from 1.01 to 1.02. However, we did not find a significant association between black carbon and cognitive impairment. The point estimates of the relative risk values did not change substantially after performing the sensitivity analyses.

**Conclusions:**

Our findings strengthen the idea that long-term exposure to PM_2.5_ mass and its chemical components is associated with an elevated risk of incident cognitive impairment among older adults.

**Supplementary Information:**

The online version contains supplementary material available at 10.1007/s40520-024-02793-9.

## Introduction

Dementia is a major public health issue with a massive social and economic burdens worldwide [1]. According to a national cross-sectional study conducted between 2015 and 2018, an estimated 6.0% of older adults aged ≥ 60 years had prevalent dementia, and the number of dementia cases was estimated at 15.07 million [2]; the number is expected to increase to 48.98 million by 2050 [3]. Since there is no curative treatment, early prevention is an effective strategy for slowing the rise in the incidence and prevalence of dementia [4].

Cognitive impairment progresses from mild to severe stages [5, 6]. Mild cognitive impairment, also known as cognitive impairment without dementia, is an important stage in which early intervention can prevent dementia [7, 8]. While the genetic factors recognized as significant risk factors for cognitive impairment and dementia [9], a significant part of cognitive impairment and dementia cannot be explained by genetic factors. Environmental risk factors may account for some of the unexplained risks [10, 11], including fine particular matter (PM_2.5_). A recent study showed that an increase in PM_2.5_ concentrations was associated with a slight increase in dementia risk [12].

PM_2.5_ contains several chemical components, including sulfate (SO_4_^2−^), nitrate (NO_3_^−^), ammonium (NH_4_^+^), organic matter (OM), and black carbon (BC). According to previous epidemiological research, long-term exposure to higher concentrations of PM_2.5_ and its components may increase the risk of mortality [13].

However, limited information is available regarding the relationship between the chemical components of PM_2.5_ and cognitive function. Pan et al., in a cross-sectional study involving 1834 participants, observed negative associations between an increase in the three PM_2.5_ components (organic carbon, potassium, iron, and ammonium ion) and cognitive decline [14]. In this study, we performed a prospective cohort study among community-dwelling older adults, estimated individual exposure to long-term PM_2.5_ and its components, and evaluated its effect on incident cognitive impairment. We aimed to identify the major components of PM_2.5_ that have an impact on cognitive function to further investigate its mechanism of action and develop a preventive strategy against dementia.

## Methods

### Study population

Study populations were derived from a community-dwelling cohort, a public health surveillance project that aims to investigate health issues among adults aged ≥ 60 years in Zhejiang province, eastern China. Details of the project can be found elsewhere [15]. Briefly, 7 of the 90 counties were chosen from Zhejiang according to population stability, exposure to potential risk factors, local disease patterns, the quality of disease and death registries, and staff capacity; at least 1500 local permanent participants aged ≥ 60 years were recruited from each county between 2014 and 2015. At baseline, each participant was invited to complete a face-to-face interview that included sociodemographic information, lifestyle, history of chronic diseases, and cognitive assessment. The participants were followed up until the end of 2020.

### Cognitive assessment

Cognitive function was evaluated using the Mini-Mental State Examination scale (MMSE). The scale has 30 items, and the total score ranges from 0 to 30, with higher total scores indicating better cognitive performance [16]. According to previous literature [17], a battery of education-specific cut-off scores was adopted to identify the individuals with cognitive impairment: ≤24 for those with higher than primary education level, ≤ 20 for those with primary education level, and ≤ 17 for illiteracy. The cognitive impairment was somewhat similar to the major and mild neurocognitive disorder in the Diagnostic and Statistical Manual of Mental Disorders (Fifth Edition), although it was not a clinical diagnosis [18].

### Estimation of residential exposure to PM_2.5_ and its constitutes

The concentrations of PM_2.5_ and its five chemical constituents (SO_4_^2−^, NO_3_^−^, NH_4_^+^, OM, and BC) were obtained from “Tracking Air Pollution in China” (TAP, http://tapdata.org.cn/) at a spatial resolution of 10 km×10 km from 2004 to 2013. Details about these data can be found in previous studies [19, 20]. Briefly, a long-term simulation was carried out to derive component-specific conversion factors based on the Weather Research and Forecasting-Community Multiscale Air Quality (WRF-CMAQ) model with an improved windblown dust module. The conversion factors were then combined with multisource-fusion PM_2.5_ data to obtain chemical component concentrations, which were then fed into machine learning models to reduce the biases. Cross validation results showed good agreement between the estimations and observations, with R^2^ values of 0.75 for NO_3_^−^, 0.70 for SO_4_^2−^, 0.75 for NH_4_^+^, 0.64 for BC, and 0.72 for OM. The TAP data have been validated and utilized in several observational epidemiological studies [21, 22]. Following previous studies [23, 24], we calculated the mean exposure concentrations at 1, 2, 3, 4, 5, and 10 years prior to the baseline enrollment for each participant at their residential addresses.

### Covariates

This study included the following acceptable or potential confounders from previous studies [25, 26]: age, sex, body mass index (BMI), education level, financial status, smoking status, drinking status, physical exercise, hypertension, hyperlipidemia, diabetes, depression, Parkinson’s disease, stroke, and family history of dementia. Financial status was self-reported by each participant, including poor, median, and rich.

### The follow-up of cohort

We re-interviewed survivors from the baseline cohort in 2015–2016 and 2019–2020 using the same questionnaire. After excluding 1525 participants with baseline cognitive impairment or incomplete cognitive assessment, 1571 lost to the follow-up or death, and one with missing values in key variables, 7804 remained for further follow-up data analyses. The MMSE identified 3249 participants who developed incident cognitive impairment.

### Statistical analysis

We conducted descriptive analyses of the individual characteristics and compared the participants with and without incident cognitive impairment using chi-square tests or t-tests, as appropriate. Considering the severe multicollinearity within PM_2.5_ and its components (Table S1), we used single-component robust Poisson regression models for analyses, which only included one PM_2.5_ component, to avoid the potential influences on the regression coefficient estimates [27, 28]. We estimated the relative risks (RRs) and 95% confidence intervals (CIs) describing the associations between exposure to the five chemical constituents and incident cognitive impairment in individuals who were free from cognitive impairment at baseline [29]. We conducted a set of sensitivity analyses to verify the robustness of our results. First, we tried different covariates for adjustment in the models: Model 1 was adjusted for age and sex; Model 2 was additionally adjusted for education and financial status; Model 3 was additionally adjusted for BMI, physical exercise, smoking status, and drinking status; and Model 4 was additionally adjusted for hypertension, hyperlipidemia, diabetes, depression, Parkinson’s disease, stroke, and a family history of dementia. Second, we used different exposure periods for the analyses, including 1–10 years before the baseline survey. Finally, we applied another model that could estimate the RR, log-binomial regression, for the analyses. All statistical analyses were conducted by SAS software (version 9.4; SAS, Cary, NC, USA). Statistical significance was defined as a two-tailed *p*-value < 0.05.

## Results

This study involved a total of 7804 participants, of which 3249 developed cognitive impairment during follow-up, with an incidence rate of 89.7/1000 person-years. The average age was 68.2 (SD 6.7) years, 52.5% were women, 10.4% had BMI of ≥ 28.0 kg/m^2^, 46.9% were illiterate, 9.7% reported poor financial status, 19.3% did physical exercise, 20.5% were current smokers, 26.9% were current drinkers, 43.9% had hypertension, and 0.6% had a family history of dementia. Individuals with cognitive impairment tended to be older, have a higher percentage of females and illiteracy, were less physically active, were more likely to having hypertension, depression, and stroke, and less likely to have hyperlipidemia and diabetes. Moreover, there were differences in the distribution of financial status, BMI, smoking status, and drinking status. Additional details are provided in Table 1. The mean individual 10-year exposure concentrations of PM_2.5_, SO_4_^2−^, NO_3_^−^, NH_4_^+^, OM, and BC prior to the baseline enrollment were 50.61, 10.41, 9.56, 7.36, 11.61, and 2.75 µg/m^3^, respectively (Table S2).

**Table 1:** Baseline characteristics of the enrolled participants

Characteristics	Total (*n* = 7804)	Cognitive impairment		*P*
		No (*n* = 4555)	Yes (*n* = 3249)	
Age	68.2 (6.7)	66.6 (5.8)	70.3 (7.3)	< 0.001^*^
Body mass index (kg/m^2^)				
<18.5	371 (4.8)	155 (3.4)	216 (6.7)	< 0.001^*^
18.5–23.9	4001 (51.3)	2259 (49.6)	1742 (5.6)	
24.0-27.9	2622 (33.6)	1627 (35.7)	995 (30.6)	
≥28.0	810 (10.4)	514 (11.3)	296 (9.1)	
Women (%)	4096 (52.5)	2272 (49.9)	1824 (56.1)	< 0.001^*^
Illiteracy (%)	3661 (46.9)	2007 (44.1)	1654 (50.9)	< 0.001^*^
Financial status (%)				
Rich	753 (9.7)	499 (11.0)	254 (7.8)	< 0.001^*^
Median	6301 (80.7)	3694 (81.1)	2607 (80.2)	
Poor	750 (9.6)	362 (8.0)	388 (11.9)	
Physical exercise (%)	1504 (19.3)	958 (21.0)	546 (16.8)	< 0.001^*^
Smoking status (%)				
Never smokers	5451 (69.9)	3129 (68.7)	2322 (71.5)	0.021^*^
Ex-smokers	752 (9.6)	960 (21.1)	641 (19.7)	
Current smokers	1601 (20.5)	466 (10.2)	286 (8.8)	
Drinking status (%)				
Never drinkers	5100 (65.4)	2918 (64.1)	2182 (67.2)	< 0.001^*^
Ex-drinkers	604 (7.7)	334 (7.3)	270 (8.3)	
Current drinkers	2100 (26.9)	1303 (28.6)	797 (24.5)	
Hypertension (%)	3426 (43.9)	1945 (42.7)	1481 (45.6)	0.011^*^
Hyperlipidemia (%)	479 (6.1)	329 (7.2)	150 (4.6)	< 0.001^*^
Diabetes (%)	693 (8.9)	431 (9.5)	262 (8.1)	0.032^*^
Depression (%)	586 (7.5)	303 (6.7)	283 (8.7)	< 0.001^*^
Parkinson’s disease (%)	21 (0.3)	9 (0.2)	12 (0.4)	0.149
Stroke (%)	179 (2.3)	75 (1.7)	104 (3.2)	< 0.001^*^
Family history of dementia (%)	49 (0.6)	30 (0.7)	19 (0.6)	0.684

^*^ Statistically significant

The 10-year exposure concentrations of PM_2.5_ and its five chemical constituents for the individuals with and without incident cognitive impairment are presented in Table 2. Individuals with incident cognitive impairment had slightly higher exposures to PM_2.5_, SO_4_^2−^, NO_3_^−^, and NH_4_^+^. The differences in PM_2.5_ and NO_3_^−^ exposure between the two groups were statistically significant, and the difference in NH_4_^+^ exposure was close to being significant.

**Table 2:** Ten years exposure to PM_2.5_ and its components in participants with and without incident cognitive impairment

Exposure (µg/m^3^)	Cognitive impairment		*P*
	No	Yes	
PM_2.5_	50.2 (13.3)	51.2 (14.2)	0.002^*^
Sulfate	10.4 (2.6)	10.5 (2.8)	0.224
Nitrate	9.5 (3.2)	9.7 (3.3)	0.010^*^
Ammonium	7.3 (2.0)	7.4 (2.1)	0.062
Organic matter	11.6 (1.9)	11.6 (2.1)	0.347
Black carbon	2.8 (0.4)	2.7 (0.4)	0.121

^*^ Statistically significant

**Fig. 1:**
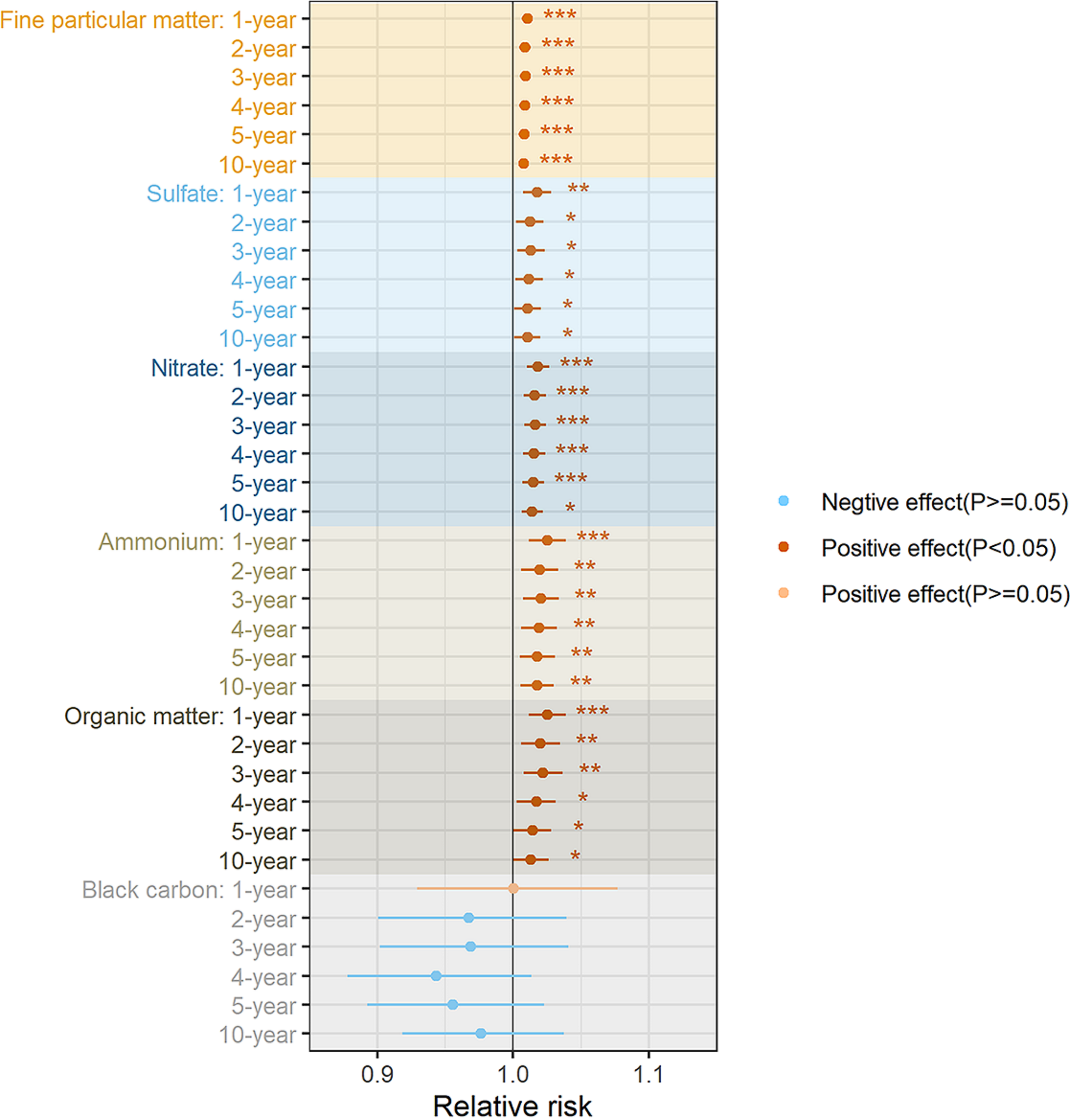
Associations between PM_2.5_ and its components with cognitive impairment in older people across exposure periods Notes: The relative risks of PM_2.5_ means the increased risk for cognitive impairment with every 2 µg/m^3^ increment. The RRs of five chemical components (sulfate, nitrate, ammonium, organic matter, and black carbon) means the increased risk for cognitive impairment with every 1 µg/m^3^ increment

The associations of 10-year exposure to PM_2.5_ and its five chemical components with incident cognitive impairment in older adults are demonstrated in Table 3. A rise of 2 µg/m^3^ in PM_2.5_ was associated with a 1% higher risk of cognitive impairment when age and sex were adjusted. A 1 µg/m^3^ increase in 10-year SO_4_^2−^, NO_3_^−^ NH_4_^+^, and OM exposures was associated with a 1%, 1%, 2%, and 1% higher risk of cognitive impairment, respectively, and all the associations were statistically significant. The associations between SO_4_^2−^, NO_3_^−^ NH_4_^+^, and OM and incident cognitive impairment across different exposure periods were similar. Further details are provided in Fig. 1 and Table S4. BC was not significantly associated with cognitive impairment after adjusting for socioeconomic variables, lifestyles, and comorbidities.

**Table 3:** Associations of 10-year exposure of PM_2.5_ and its components with incident cognitive impairment in older people

Pollutants	Models	RR	95% CI	*P*
PM_2.5_	Model 1	1.00	1.00-1.01	0.003^*^
	Model 2	1.00	1.00-1.01	< 0.001^*^
	Model 3	1.00	1.00-1.01	< 0.001^*^
	Model 4	1.00	1.00-1.01	< 0.001^*^
Sulfate	Model 1	1.01	1.00-1.02	0.271
	Model 2	1.01	1.00-1.02	0.134
	Model 3	1.01	1.00-1.02	0.046^*^
	Model 4	1.01	1.00-1.02	0.032^*^
Nitrate	Model 1	1.01	1.00-1.02	0.012^*^
	Model 2	1.01	1.01–1.02	0.001^*^
	Model 3	1.01	1.01–1.02	0.001^*^
	Model 4	1.01	1.01–1.02	0.001^*^
Ammonium	Model 1	1.01	1.00-1.03	0.073
	Model 2	1.02	1.00-1.03	0.014^*^
	Model 3	1.02	1.00-1.03	0.008^*^
	Model 4	1.02	1.01–1.03	0.005^*^
Organic matter	Model 1	1.01	0.99–1.02	0.430
	Model 2	1.01	1.00-1.02	0.175
	Model 3	1.01	1.00-1.03	0.060
	Model 4	1.01	1.00-1.03	0.045^*^
Black carbon	Model 1	0.94	0.89–1.01	0.078
	Model 2	0.94	0.89-1.00	0.058
	Model 3	0.97	0.91–1.03	0.355
	Model 4	0.98	0.92–1.04	0.439

Model 1: adjusted for age and sex. Model 2: Model 1 + education and financial status. Model 3: Model 2 + BMI, physical exercise, smoking status and drinking status. Model 4: adjusted for Model 3 + hypertension, hyperlipidemia, diabetes, depression, Parkinson’s disease, stroke, and family history of dementia.

^*^ Statistically significant.

Our results were robust in a battery of sensitivity analyses. First, the relative effect estimates did not change and remained statistically significant when different covariates (age and sex [model 1], socioeconomic variables [model 2], lifestyles [model 3], and co-morbidities [model 4]) were taken into account (Table 3). Second, the impact of PM_2.5_ and its five chemical constituents on incident cognitive impairment risk did not vary significantly by exposure period (Table S4). This indicates that the effect amy not be influenced by the exposure period. Lastly, the point estimates of the RR values did not change substantially after performing the analyses using the alternative model (log-binomial regression), except for the *P*-value, which was affected by the non-convergence of the algorithm. And similarly, the relative effect estimates did not change substantially across different exposure periods (Table S5).

## Discussion

Our community-dwelling cohort study investigated the effect of PM_2.5_ and its five chemical constituents on cognitive impairment among older adults. The findings indicated that there was a positive association between PM_2.5_ and cognitive impairment. Higher exposure to SO_4_^2−^, NO_3_^−^, NH_4_^+^, and OM, but not in BC, was associated with an increased risk of cognitive impairment.

Our results are consistent with the findings from earlier literature regarding the relationship between PM_2.5_ and cognitive impairment. Findings from the Swedish National Study on Aging and Care in Kungsholmen (SNAC-K) indicated that an increase in PM_2.5_ exposure of 1 µg/m^3^ was associated with a 75% higher risk of incident cognitive impairment (hazard ratio [HR] = 1.75, 95% CI: 1.54–1.99) [30]. A cohort study from Ontario, Canada reported that PM_2.5_ was positively associated with dementia, and every increase in PM_2.5_ exposure over the interquartile range (4.8 µg/m^3^) corresponded to a 4% higher risk of incident dementia (HR = 1 0.04, 95% CI: 1.03–1.05) [31]. A national cohort study from the United States reported that per interquartile range increase in 5-year PM_2.5_ exposure (3.2 µg/m^3^) corresponded to a 6% higher risk for incident dementia (HR = 1.06, 95% CI: 1.05–1.07) [32]. In China, a cohort from Hong Kong also reported a positive association between PM_2.5_ exposure and incident dementia, with an HR of 1.06 (95% CI: 1.00-1.13) for every 3.8 µg/m^3^ increase in PM_2.5_ exposure [33]. And Yan et al. reported that the dementia risk increases with every 10.3 µg/m^3^ increase of PM_2.5_ (HR = 1.05, 95% CI: 1.04–1.05) [34].

Fewer studies have investigated the association between the components of PM_2.5_ and cognitive impairment. Recently, Shi et al. analyzed databases from the Centers for Medicare and Medicaid Services and reported that incident dementia was significantly associated with the interquartile range levels of BC, OM, SO_4_^2−^, and NH_4_^+^. The HRs (95% CIs) of dementia were 1.04 (95% CI: 1.04–1.05) for BC, 1.04 (95% CI: 1.03–1.04) for OM, 1.11 (95% CI: 1.10–1.11) for SO_4_^2−^, 1.07 (95% CI: 1.06–1.07) for NH_4_^+^, and 1.01 (95% CI: 1.00-1.01) for NO_3_^−^ for every interquartile range increase in each pollutant [27]. In this study, we found that the risk of cognitive impairment significantly increased in older adults with higher exposure to SO_4_^2−^, NO_3_^−^, NH_4_^+^, and OM, but not BC. The inconsistencies in the findings could result from the discrepancies in the study population, exposure level to PM_2.5_ and its components, algorithms for estimating PM_2.5_ and its component concentrations, and covariates adjustments.

Studies regarding BC and cognitive function are limited. Two studies from the United States have indicated that BC is positively associated with cognitive decline [27, 35]. However, the association between elemental carbon and cognitive function was not statistically significant according to the findings of a study from China [14]. Further studies are required to confirm this hypothesis.

NH_4_^+^ is a neurotoxic agent that can damage the blood-brain barrier structure [36, 37]. Experimental and observational studies have indicated an potential association between NH_4_^+^ and cognitive function [38, 39]. NH_4_^+^ in PM_2.5_ is mostly contributed by agricultural (such as fertilizer application and livestock excreta) and non-agricultural (such as fossil fuel combustion) activities, and the predominant sources may have been non-agricultural activities in our study [40–43].

The impact of SO_4_^2−^ could be explained by oxidative stress. SO_4_^2−^ can reduce the pH of aerosols and thus solubilize metals that contribute to reactive oxygen species (ROS). Oxidative stress in the body follows the exceeding concentration of ROS [44]. Increased oxidative stress is associated with the formulation and accumulation of amyloid-β (Aβ) and hyperphosphorylated Tau protein (pTau), which could represent a vicious cycle, eventually leading to neuronal death [45].

NH_4_NO_3_ is the main form of nitrates in PM_2.5_ [46]. NO_3_^−^ is a potential reactive nitrogen species (RNS) that functions in signal transduction pathways, and contributes to the immune responses by functioning as a nonspecific defense at appropriate concentrations. However, excess RNS may induce cellular damage in oxidative environments [47].

OM constituted a large fraction of PM_2.5_ mass in our study. OM is composed of primary OM from combustion emissions and direct emissions from other sources, as well as secondary OM formed when gas-phase species are oxidized in the atmosphere [48]. The components of OM are complexity; highly toxic species, such as polycyclic aromatic hydrocarbons and polychlorinated biphenyls, are always found in OM [49, 50], which are associated with an increased risk of dementia [51, 52].

Our study had some limitations. First, the exposure assessment was at a relatively coarse spatial scale, which may bias the estimates of individual exposure doses. However, considering the distance range of daily activities of individuals around their residential address, the assessment results in this study are also representative of the PM_2.5_ exposure levels of the participants. Second, although all the participants were local permanent residents, some participants would sometimes work away from home, and our study did not record these residential changes. Thus, the exposure concentrations estimated in this study may have differed from the actual concentrations. This may also lead to a bias in the precision of the individual exposure dose estimates. Third, we did not consider the effects of indoor pollution, which could have affected our estimates. Finally, cognitive impairment in this study was assessed using the MMSE scale rather than a clinical or experimental diagnosis, which may lead to an inaccurate evaluation of cognitive function. Therefore, caution should be exercised when interpreting our findings. In the future, more precise methods for estimating air pollutant exposure and more accurate tools for assessing cognitive function in community-dwelling older adults should be considered.

## Conclusion

Overall, this study observed a positive association between PM_2.5_ and cognitive impairment and found that higher exposure to SO_4_^2−^, NO_3_^−^, NH_4_^+^, and OM in PM_2.5_, was associated with an increased risk of cognitive impairment. These results strengthen the idea that long-term exposure to PM_2.5_ and its five chemical constituents is associated with an increased risk of incident cognitive impairment among older adults. Most importantly, our findings indicate that limiting and reducing PM_2.5_ emission sources, such as the burning of fossil fuels, could have considerable public health benefits in the current context of global aging.

### Electronic supplementary material

Below is the link to the electronic supplementary material.

Supplementary Material 1

## Data Availability

Due to containing sensitive information, data are available from the Ethics Committee of Zhejiang Provincial Center for Disease Control and Prevention (http://www.cdc.zj.cn/, contact via Zhengting Wang, ztwang@cdc.zj.cn) for researchers who meet the criteria for accessing confidential data.
