# Supplementary material for: Association between ambient fine particular matter components and subsequent cognitive impairment in community-dwelling older people: a prospective cohort study from eastern China
Source: Aging Clin Exp Res. 2024 Jul 26;36(1):150. doi: 10.1007/s40520-024-02793-9 (PMC11282123; doi:10.1007/s40520-024-02793-9)
Supplement: Supplementary file 1 — Supplementary Material 1 [file 40520_2024_2793_MOESM1_ESM.docx]

**Association between Ambient Fine Particular Matter Components and Subsequent Cognitive Impairment in Community-Dwelling Older People: a Prospective Cohort Study from Eastern China**

Tao Zhang^1^**^†^**, Wenfeng Liu^2^**^†^**, Tao Yang^3^**^†^**, Yujia Zhai^1^, Xue Gu^1^, Le Xu^1^, Fudong Li^1^, Mengna Wu^1^, Junfen Lin^1^

^1^ Department of Public Health Surveillance and Advisory, Zhejiang Provincial Center for Disease Control and Prevention, Hangzhou, Zhejiang, China

^2^ Office, Changshan Center for Disease Control and Prevention, Quzhou, Zhejiang, China

^3^ Office, Yuhang Center for Disease Control and Prevention, Hangzhou, Zhejiang, China

**^†^ These authors contributed equally to this work and share first authorship**

**^*^ Correspondence:** Junfen Lin, +86-571-87115131, 1051681514@qq.com

Table S1 Pearson correlation coefficients within 10-year average exposure concentrations of PM_2.5_ and its components

|  | PM_2.5_ | Sulfate | Nitrate | Ammonium | Organic matter | Black carbon |
| --- | --- | --- | --- | --- | --- | --- |
| PM_2.5_ | 1.000 | 0.987 | 0.994 | 0.990 | 0.985 | 0.937 |
| Sulfate |  | 1.000 | 0.989 | 0.990 | 0.990 | 0.974 |
| Nitrate |  |  | 1.000 | 0.998 | 0.979 | 0.933 |
| Ammonium |  |  |  | 1.000 | 0.984 | 0.941 |
| Organic matter |  |  |  |  | 1.000 | 0.971 |
| Black carbon |  |  |  |  |  | 1.000 |

Table S2 Statistical description of the exposure concentrations of PM_2.5_ and its constituents and meteorological parameters on individual level

| Exposure (μg/m^3^) | Mean | SD | Min | Median | Max | IQR |
| --- | --- | --- | --- | --- | --- | --- |
| 10-year mean exposure |  |  |  |  |  |  |
| PM_2.5_ | 50.61 | 13.70 | 24.72 | 51.99 | 69.65 | 27.34 |
| Sulfate | 10.41 | 2.66 | 5.41 | 11.04 | 13.57 | 5.29 |
| Nitrate | 9.56 | 3.23 | 4.17 | 9.27 | 13.80 | 7.03 |
| Ammonium | 7.36 | 2.07 | 3.68 | 6.99 | 9.98 | 4.44 |
| Organic matter | 11.61 | 1.98 | 7.01 | 11.73 | 14.05 | 3.51 |
| Black carbon | 2.75 | 0.41 | 1.80 | 2.93 | 3.29 | 0.72 |
| 5-year mean exposure |  |  |  |  |  |  |
| PM_2.5_ | 48.26 | 12.80 | 23.46 | 50.26 | 65.08 | 25.98 |
| Sulfate | 9.96 | 2.59 | 5.08 | 10.45 | 12.97 | 5.33 |
| Nitrate | 9.66 | 3.21 | 4.20 | 9.32 | 13.81 | 6.93 |
| Ammonium | 7.20 | 2.00 | 3.60 | 6.85 | 9.66 | 4.27 |
| Organic matter | 10.83 | 1.86 | 6.49 | 10.93 | 13.00 | 3.50 |
| Black carbon | 2.48 | 0.37 | 1.62 | 2.63 | 2.94 | 0.64 |
| 1-year exposure |  |  |  |  |  |  |
| PM_2.5_ | 47.14 | 12.35 | 23.80 | 47.70 | 65.00 | 22.30 |
| Sulfate | 9.65 | 2.46 | 5.11 | 10.00 | 12.80 | 4.93 |
| Nitrate | 9.66 | 3.14 | 4.39 | 9.13 | 14.00 | 6.29 |
| Ammonium | 7.04 | 1.90 | 3.63 | 6.55 | 9.58 | 3.71 |
| Organic matter | 10.74 | 1.91 | 6.58 | 10.72 | 13.62 | 2.91 |
| Black carbon | 2.35 | 0.35 | 1.60 | 2.36 | 2.78 | 0.73 |

Table S3 Exposure to PM_2.5_ and its components prior to the baseline enrollment in participants with and without cognitive impairment

| Exposure (μg/m^3^) | Cognitive impairment | | *P* |
| --- | --- | --- | --- |
|  | No | Yes |  |
| 5-year mean exposure |  |  |  |
| PM_2.5_ | 47.9 (12.5) | 48.8 (13.2) | 0.004 |
| Sulfate | 9.9 (2.5) | 10.0 (2.7) | 0.270 |
| Nitrate | 9.6 (3.1) | 9.8 (3.3) | 0.008 |
| Ammonium | 7.2 (2.0) | 7.2 (2.1) | 0.076 |
| Organic matter | 10.8 (1.8) | 10.9 (1.9) | 0.390 |
| Black carbon | 2.5 (0.4) | 2.5 (0.4) | 0.038 |
| 1-year exposure |  |  |  |
| PM_2.5_ | 46.7 (11.9) | 47.8 (12.9) | <0.001 |
| Sulfate | 9.6 (2.4) | 9.7 (2.6) | 0.016 |
| Nitrate | 9.6 (3.1) | 9.8 (3.3) | 0.001 |
| Ammonium | 7.0 (1.8) | 7.1 (2.0) | 0.006 |
| Organic matter | 10.7 (1.8) | 10.8 (2.0) | 0.006 |
| Black carbon | 2.4 (0.3) | 2.3 (0.4) | 0.661 |

Table S4 Associations of PM_2.5_ and its components with cognitive impairment in older people estimated by robust Poisson regressions

| Pollutants | Exposure periods | RR | 95% CI | *P* |
| --- | --- | --- | --- | --- |
| Per 2μg/m^3^ increment |  |  |  |  |
| PM_2.5_ | 1-year | 1.01 | 1.01-1.01 | <0.001 |
|  | 2-year | 1.01 | 1.00-1.01 | <0.001 |
|  | 3-year | 1.01 | 1.00-1.01 | <0.001 |
|  | 4-year | 1.01 | 1.00-1.01 | <0.001 |
|  | 5-year | 1.01 | 1.00-1.01 | <0.001 |
|  | 10-year | 1.01 | 1.00-1.01 | <0.001 |
| Per 1μg/m^3^ increment |  |  |  |  |
| Sulfate | 1-year | 1.02 | 1.01-1.03 | 0.001 |
|  | 2-year | 1.01 | 1.00-1.02 | 0.017 |
|  | 3-year | 1.01 | 1.00-1.02 | 0.011 |
|  | 4-year | 1.01 | 1.00-1.02 | 0.025 |
|  | 5-year | 1.01 | 1.00-1.02 | 0.038 |
|  | 10-year | 1.01 | 1.00-1.02 | 0.032 |
| Nitrate | 1-year | 1.02 | 1.01-1.03 | <0.001 |
|  | 2-year | 1.02 | 1.01-1.02 | <0.001 |
|  | 3-year | 1.02 | 1.01-1.02 | <0.001 |
|  | 4-year | 1.02 | 1.01-1.02 | <0.001 |
|  | 5-year | 1.01 | 1.01-1.02 | <0.001 |
|  | 10-year | 1.01 | 1.01-1.02 | 0.001 |
| Ammonium | 1-year | 1.03 | 1.01-1.04 | <0.001 |
|  | 2-year | 1.02 | 1.01-1.03 | 0.004 |
|  | 3-year | 1.02 | 1.01-1.03 | 0.002 |
|  | 4-year | 1.02 | 1.01-1.03 | 0.004 |
|  | 5-year | 1.02 | 1.01-1.03 | 0.006 |
|  | 10-year | 1.02 | 1.01-1.03 | 0.005 |
| Organic matter | 1-year | 1.03 | 1.01-1.04 | <0.001 |
|  | 2-year | 1.02 | 1.01-1.03 | 0.005 |
|  | 3-year | 1.02 | 1.01-1.04 | 0.002 |
|  | 4-year | 1.02 | 1.00-1.03 | 0.020 |
|  | 5-year | 1.01 | 1.00-1.03 | 0.042 |
|  | 10-year | 1.01 | 1.00-1.03 | 0.045 |
| Black carbon | 1-year | 1.00 | 0.93-1.08 | 0.993 |
|  | 2-year | 0.97 | 0.90-1.04 | 0.364 |
|  | 3-year | 0.97 | 0.90-1.04 | 0.387 |
|  | 4-year | 0.94 | 0.88-1.01 | 0.110 |
|  | 5-year | 0.96 | 0.89-1.02 | 0.190 |
|  | 10-year | 0.98 | 0.92-1.04 | 0.439 |

Table S5 Associations of PM_2.5_ and its components with cognitive impairment in older people estimated by log-binomial regressions

|  | Exposure periods | RR | 95%CI | P |
| --- | --- | --- | --- | --- |
| Per 2μg/m^3^ increment |  |  |  |  |
| PM_2.5_ | 1-year | 1.01 | 1.00-1.02 | 0.003 |
|  | 2-year | 1.01 | 1.00-1.02 | 0.010 |
|  | 3-year | 1.01 | 1.00-1.02 | 0.008 |
|  | 4-year | 1.01 | 1.00-1.02 | 0.012 |
|  | 5-year | 1.01 | 1.00-1.02 | 0.014 |
|  | 10-year | 1.01 | 1.00-1.01 | 0.011 |
| Per 1μg/m^3^ increment |  |  |  |  |
| Sulfate | 1-year | 1.02 | 1.00-1.04 | 0.038 |
|  | 2-year | 1.01 | 1.00-1.03 | 0.145 |
|  | 3-year | 1.01 | 1.00-1.03 | 0.125 |
|  | 4-year | 1.01 | 0.99-1.03 | 0.175 |
|  | 5-year | 1.01 | 0.99-1.03 | 0.205 |
|  | 10-year | 1.01 | 0.99-1.03 | 0.185 |
| Nitrate | 1-year | 1.02 | 1.01-1.03 | 0.007 |
|  | 2-year | 1.02 | 1.00-1.03 | 0.022 |
|  | 3-year | 1.02 | 1.00-1.03 | 0.017 |
|  | 4-year | 1.02 | 1.00-1.03 | 0.023 |
|  | 5-year | 1.02 | 1.00-1.03 | 0.029 |
|  | 10-year | 1.01 | 1.00-1.03 | 0.034 |
| Ammonium | 1-year | 1.03 | 1.00-1.05 | 0.026 |
|  | 2-year | 1.02 | 1.00-1.04 | 0.088 |
|  | 3-year | 1.02 | 1.00-1.04 | 0.070 |
|  | 4-year | 1.02 | 1.00-1.04 | 0.091 |
|  | 5-year | 1.02 | 1.00-1.04 | 0.103 |
|  | 10-year | 1.02 | 1.00-1.04 | 0.093 |
| Organic matter | 1-year | 1.03 | 1.00-1.05 | 0.023 |
|  | 2-year | 1.02 | 1.00-1.05 | 0.090 |
|  | 3-year | 1.02 | 1.00-1.05 | 0.071 |
|  | 4-year | 1.02 | 0.99-1.04 | 0.178 |
|  | 5-year | 1.01 | 0.99-1.04 | 0.243 |
|  | 10-year | 1.01 | 0.99-1.04 | 0.235 |
| Black carbon | 1-year | 1.01 | 0.89-1.16 | 0.845 |
|  | 2-year | 0.98 | 0.86-1.11 | 0.704 |
|  | 3-year | 0.97 | 0.86-1.11 | 0.693 |
|  | 4-year | 0.95 | 0.83-1.08 | 0.415 |
|  | 5-year | 0.96 | 0.85-1.08 | 0.511 |
|  | 10-year | 0.98 | 0.88-1.09 | 0.727 |
